# IDHP Mitigates LPS-Induced Cardiomyocyte Injury via the GAS6/Axl-AMPK Axis: A Multi-Target Strategy Counteracting Inflammation, Oxidative Stress, and Apoptosis

**DOI:** 10.3390/ph18081188

**Published:** 2025-08-12

**Authors:** Junmin Chen, Yijie Wang, Xingge Li, Xiaoqing Guo, Jiayin Tian, Xiaohui Zheng, Yang Yang, Yanting Cao

**Affiliations:** 1 Xi’an Key Laboratory of Innovative Drug Research for Heart Failure, Faculty of Life Sciences and Medicine, Northwest University, 229 Taibai North Road, Xi’an 710069, China; junminchennw@163.com (J.C.); yijiewangnw@163.com (Y.W.); xinggelinw@163.com (X.L.); xiaoqingguonw@163.com (X.G.); jiayintiannw@163.com (J.T.); xiaohuizhengnw@163.com (X.Z.); 2 Department of Cardiology, Affiliated Hospital, Yan’an University, 43 North Street, Yan’an 716000, China; 3 Department of Ophthalmology, Xi’an People’s Hospital (Xi’an Fourth Hospital), 21 Jiefang Road, Xi’an 710000, China

**Keywords:** IDHP, sepsis-induced myocardial injury, GAS6/Axl-AMPK, oxidation, apoptosis

## Abstract

**Background:** Sepsis-induced myocardial injury (SIMI) significantly contributes to sepsis-related mortality, yet effective therapies remain limited. This study investigated the cardioprotective potential of isopropyl 3-(3,4-dihydroxyphenyl)-2-hydroxypropanoate (IDHP), a bioactive metabolite from *Salvia miltiorrhiza*, focusing on its mechanism via the GAS6/Axl signaling axis in lipopolysaccharide (LPS)-induced myocardial injury. **Methods:** Using an in vitro HL-1 cardiomyocyte model, IDHP’s cytotoxicity was assessed (0–20 μM). Cells were pretreated with IDHP (10 μM, optimal concentration) before LPS exposure. Inflammatory cytokines (IL-6/TNF-α/IL-1β/IL-18), chemokines (CCL2/CCR2, CCL25/CCR9), ROS levels (Nrf2 pathway), and apoptosis markers (Bax) were quantified. GAS6/Axl-AMPK signaling was evaluated via GAS6 knockout experiments. **Results:** IDHP (≤20 μM) showed no cytotoxicity. At 10 μM, it exhibited anti-inflammatory effects by reducing LPS-induced cytokine/chemokine release, demonstrated antioxidant activity through lowering ROS via Nrf2 activation, and exerted anti-apoptotic action by downregulating Bax. Mechanistically, IDHP restored GAS6/Axl-AMPK phosphorylation, an effect abolished in GAS6-knockout cells. **Conclusions:** IDHP mitigates LPS-induced cardiomyocyte injury by concurrently targeting inflammation, oxidative stress, and apoptosis via GAS6/Axl-AMPK signaling, proposing a novel therapeutic avenue for SIMI.

## 1. Introduction

Sepsis is a life-threatening organ dysfunction caused by a dysregulated host response to infection; epidemiological data indicate that approximately half of sepsis patients develop sepsis-induced myocardial injury (SIMI) [1,2]. Pathophysiologically, SIMI is characterized by clinical manifestations including ventricular remodeling, diminished pump function, and systolic dysfunction. Its pathogenesis involves multiple mechanisms, encompassing uncontrolled systemic inflammatory response, exacerbated oxidative stress damage, mitochondrial energy metabolism dysfunction, and dysregulated myocardial calcium homeostasis [3,4,5]. Although current clinical strategies combining vasoactive drugs with antibiotics have yielded certain efficacy [6], specific therapeutic approaches for SIMI remain significantly limited.

*Salvia miltiorrhiza* Bunge (Dan Shen), a traditional Chinese herb renowned for cardiovascular protection, is widely used in treating coronary artery disease and atherosclerosis [7,8]. Its active components (tanshinones and salvianolic acids) exhibit anti-inflammatory, antioxidant, and anti-apoptotic properties [9]. IDHP (isopropyl 3-(3,4-dihydroxyphenyl)-2-hydroxypropanoate), identified by Professor Zheng Xiaohui’s team among multiple *Salvia miltiorrhiza*-derived metabolites, demonstrates anti-inflammatory, anti-fibrotic, vasodilatory, anti-ischemia–reperfusion injury, and anti-aging effects in preclinical studies [10]. Prior research indicates IDHP mitigates myocardial fibrosis, reduces ischemia–reperfusion injury, and preserves vascular function through multi-target mechanisms—modulating inflammatory signaling, activating antioxidant defense systems, and regulating myocardial ion channels [11,12,13]. Based on our previous research, we established a sepsis-related myocardial injury model via cecal ligation and puncture (CLP). This systematic investigation evaluated the organ-protective effects of IDHP and its underlying molecular mechanisms. In vivo experiments confirmed that IDHP exerts significant cardioprotection against septic myocardial injury, thereby providing experimental evidence for targeted therapeutic strategies against sepsis-associated cardiac complications [14]. Nevertheless, its specific targets and molecular mechanisms in sepsis-related cardiac injury remain insufficiently explored.

The GAS6/Axl signaling pathway, a key member of the TAM receptor tyrosine kinase family, plays a central role in regulating inflammation, apoptosis, and mitochondrial homeostasis [15,16]. GAS6—a vitamin K-dependent ligand—specifically binds the Axl receptor, activating downstream pathways including AMP-activated protein kinase (AMPK) [17,18,19]. In cardiovascular pathologies, GAS6/Axl confers protection by: (1) inhibiting pro-inflammatory cytokine release (TNF-α, IL-6); (2) reducing ROS generation to alleviate oxidative stress [20,21,22]. This pathway is implicated in vascular calcification, ischemia–reperfusion injury (IRI), and septic cardiomyopathy [14,23,24]. Studies reveal suppressed GAS6 expression and diminished AMPK activity in sepsis-induced myocardial injury, while exogenous restoration of this pathway effectively improves cardiac dysfunction [25,26,27]. However, whether IDHP exerts cardioprotection in septic myocardial injury via GAS6/Axl regulation requires further investigation.

This study explored IDHP’s therapeutic potential for septic cardiomyopathy and elucidated its mechanism through the GAS6/Axl pathway. These findings not only demonstrate IDHP’s translational potential as a multi-target therapeutic agent for SIMI but also bridge traditional herbal active components with modern molecular cardiology, offering novel drug development strategies for sepsis-related cardiac complications.

## 2. Results

### 2.1. Toxicity Assessment of IDHP

**Figure 1:**
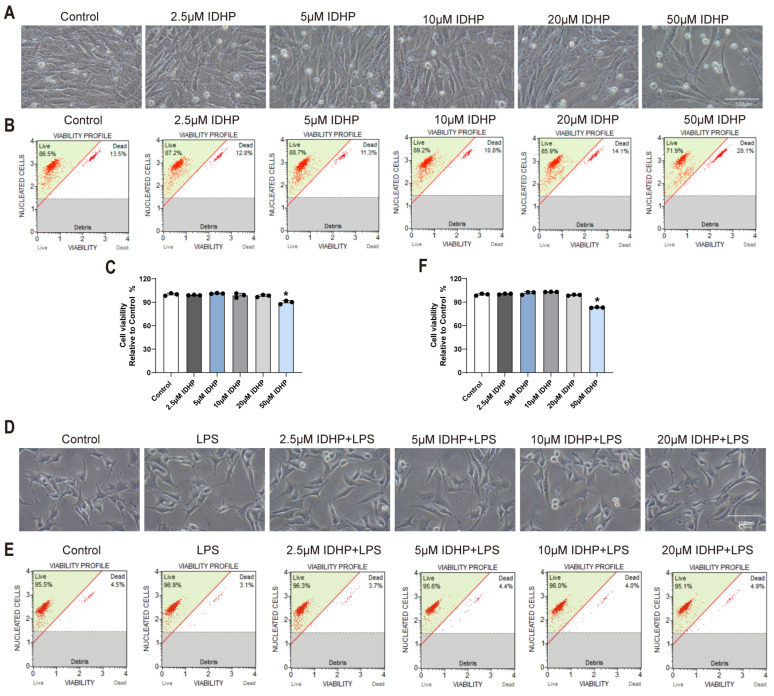
Toxicity Assessment of IDHP. (**A**) Typical morphological diagram of HL-1 cells treated by different concentrations of IDHP (2.5, 5, 10, 20 and 50 μM), n = 3. (**B**,**C**) Typical cell viability plots of HL-1 cells treated with different concentrations of IDHP (2.5, 5, 10, 20 and 50 μM) and corresponding statistical graph, n = 3. (**D**) Typical morphological diagram of HL-1 cells treated with different concentrations of IDHP (2.5, 5, 10 and 20 μM) followed by LPS injury, n = 3. (**E**,**F**) Typical cell viability plots of HL-1 cells treated with different concentrations of IDHP (2.5, 5, 10, 20 and 50 μM) followed by LPS injury and corresponding statistical graph, n = 3. * *p* < 0.05, vs. control group.

To evaluate the cellular safety of IDHP, murine cardiomyocytes were treated with gradient concentrations (2.5, 5, 10, 20, and 50 μM) for 24 h. Experimental data (Figure 1A–C) demonstrated that 50 μM IDHP significantly reduced cell viability compared to the control group, whereas concentrations ≤ 20 μM showed no significant morphological alterations or survival rate decline. Thus, 20 μM was established as the maximum safe dose for subsequent experiments. Further validation using an LPS-induced cardiomyocyte injury model (Figure 1D–F) revealed that pretreatment with 2.5–20 μM IDHP 3 h prior to LPS stimulation significantly prevented cellular damage or reduced viability compared to the LPS-only group.

### 2.2. IDHP Reverses LPS-Induced Upregulation of Pro-Inflammatory Cytokines and Chemokines

**Figure 2:**
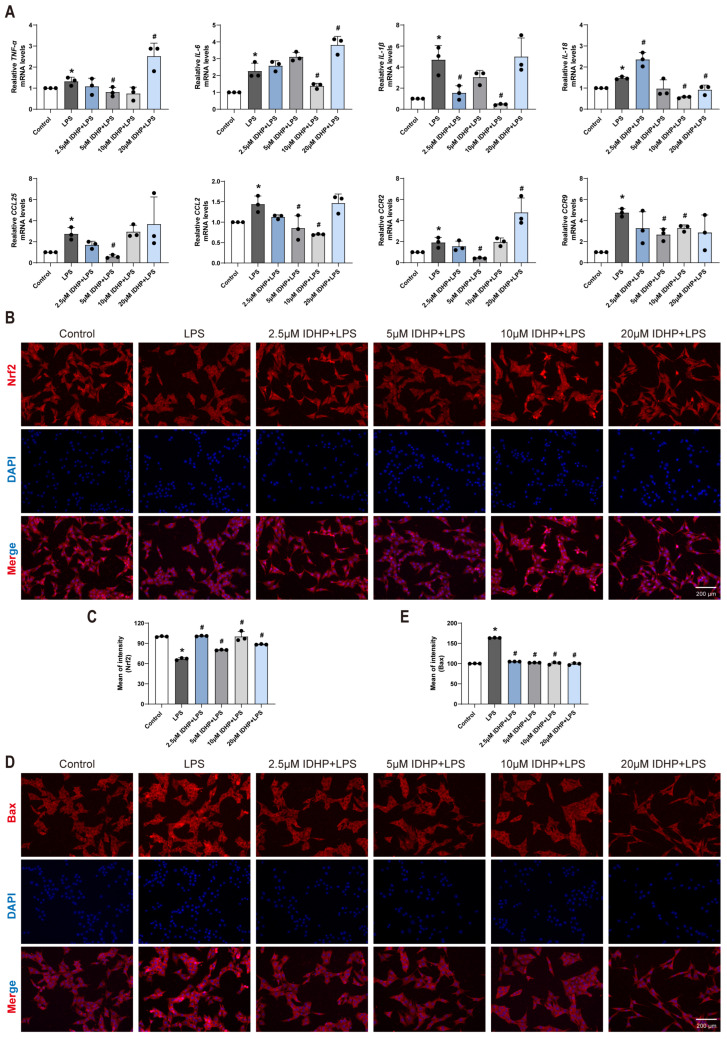
IDHP reverses LPS-induced upregulation of pro-inflammatory cytokines and chemokines while exerting cardioprotective effects by mitigating oxidative stress and inhibiting apoptosis. (**A**) Statistical graph of *TNF-α*, *IL-6*, *IL-1β*, *IL-18*, *CCL25*, *CCL2*, *CCR2* and *CCR9* mRNA analyzed by qRT-PCR in HL-1 cells, n = 3. (**B**,**C**) Immunofluorescence staining and corresponding statistical analysis of Nrf2 in HL-1 cells, n = 3. (**D**,**E**) Immunofluorescence staining and corresponding statistical analysis of Bax in HL-1 cells, n = 3. * *p* < 0.05, vs. control group; ^#^ *p* < 0.05, vs. LPS group.

qRT-PCR analysis indicated that LPS stimulation significantly elevated mRNA levels of pro-inflammatory mediators, including cytokines (TNF-α, IL-6, IL-1β, IL-18) and chemokines (CCL25, CCL2, CCR2, CCR9) (*p* < 0.05; Figure 2A). Notably, IDHP treatment significantly reversed the expression of these inflammatory markers. Among tested concentrations, 10 μM IDHP exhibited the most comprehensive anti-inflammatory effects, markedly reducing pro-inflammatory cytokine levels and suppressing chemokine signaling, thereby effectively mitigating LPS-induced cellular injury.

### 2.3. IDHP Exerts Cardioprotective Effects by Alleviating Oxidative Stress and Inhibiting Apoptosis in Cardiomyocytes

**Figure 3:**
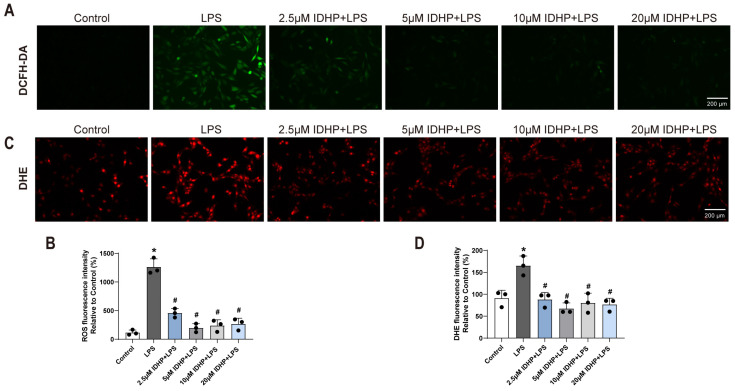

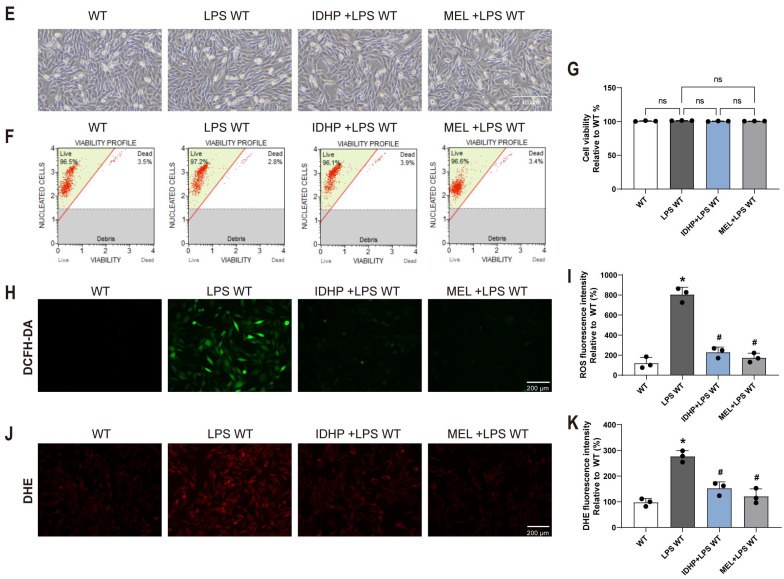
IDHP exerts cardioprotective effects by alleviating oxidative stress and inhibiting apoptosis in cardiomyocytes. (**A**,**B**) DCFH-DA staining and statistical graph in HL-1 cells, n = 3. (**C**,**D**) DHE staining and statistical plots in HL-1 cells, n = 3. * *p* < 0.05, vs. control group; ^#^ *p* < 0.05, vs. LPS group. (**E**) Typical morphological diagram of HL-1 cells in different groups, n = 3. (**F**,**G**) Typical cell viability plots of HL-1 cells in different groups and statistical graph, n = 3. ns: no significant. (**H**,**I**) DCFH-DA staining and statistical plots in HL-1 cells, n = 3. (**J**,**K**) DHE staining and statistical plots in HL-1 cells, n = 3. * *p* < 0.05, vs. WT group; ^#^ *p* < 0.05, vs. LPS WT group.

Experimental results demonstrated that compared to the control group, LPS stimulation significantly induced oxidative stress and activated apoptotic signaling pathways in cardiomyocytes. Specific manifestations included a significant decrease in the expression level of the transcription factor Nrf2 (*p* < 0.05, Figure 2B,C), indicating a diminished capacity for intracellular ROS clearance; a marked increase in the expression level of the pro-apoptotic protein Bax (*p* < 0.05, Figure 2D,E), suggesting enhanced cellular apoptosis. DHE and DCFH-DA fluorescence staining further confirmed a significant elevation in intracellular ROS levels in the LPS-treated group compared to the control group (Figure 3A–D). Notably, IDHP treatment exhibited significant protective effects by (1) restoring Nrf2 protein expression across all tested concentrations (*p* < 0.05), (2) reversing the LPS-induced overexpression of Bax protein (*p* < 0.05), and (3) reducing the accumulation of intracellular ROS (*p* < 0.05). These results substantiate, at the molecular level, that IDHP exerts its cardioprotective effects by mitigating oxidative stress and inhibiting apoptosis. Melatonin (MEL) is recognized as an effective positive control drug against sepsis. Based on this, this study employed MEL (100 μM) as a positive control to evaluate the protective effects of IDHP in an LPS-induced HL-1 cardiomyocyte injury model. The experimental results demonstrated that IDHP (10 μM) exhibited effects comparable to MEL. Cellular morphology and viability assays indicated that neither IDHP nor MEL treatment significantly impacted cardiomyocyte morphology or viability (Figure 3E–G). Subsequently, ROS detection using DHE and DCFH-DA fluorescent probes revealed that both IDHP and MEL treatments significantly reduced DHE fluorescence intensity compared to the LPS-injured group, (Figure 3H–K), indicating that both of them effectively inhibit excessive accumulation of ROS.

### 2.4. IDHP Ameliorates LPS-Induced Cardiomyocyte Injury via Activation of the GAS6/Axl-AMPK Signaling Axis

**Figure 4:**
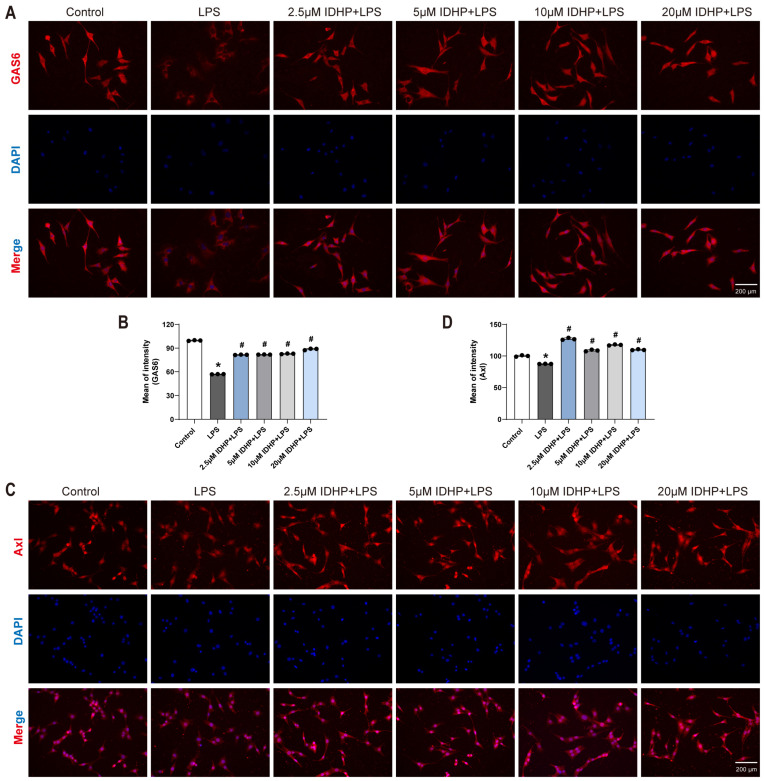
IDHP ameliorates LPS-induced cardiomyocyte injury via activation of the GAS6/Axl signaling axis. (**A**,**B**) Immunofluorescence staining and corresponding statistical analysis of GAS6 in HL-1 cells, n = 3. (**C**,**D**) Immunofluorescence staining and corresponding statistical analysis of Axl in HL-1 cells, n = 3. * *p* < 0.05, vs. control group; ^#^ *p* < 0.05, vs. LPS group.

**Figure 5:**
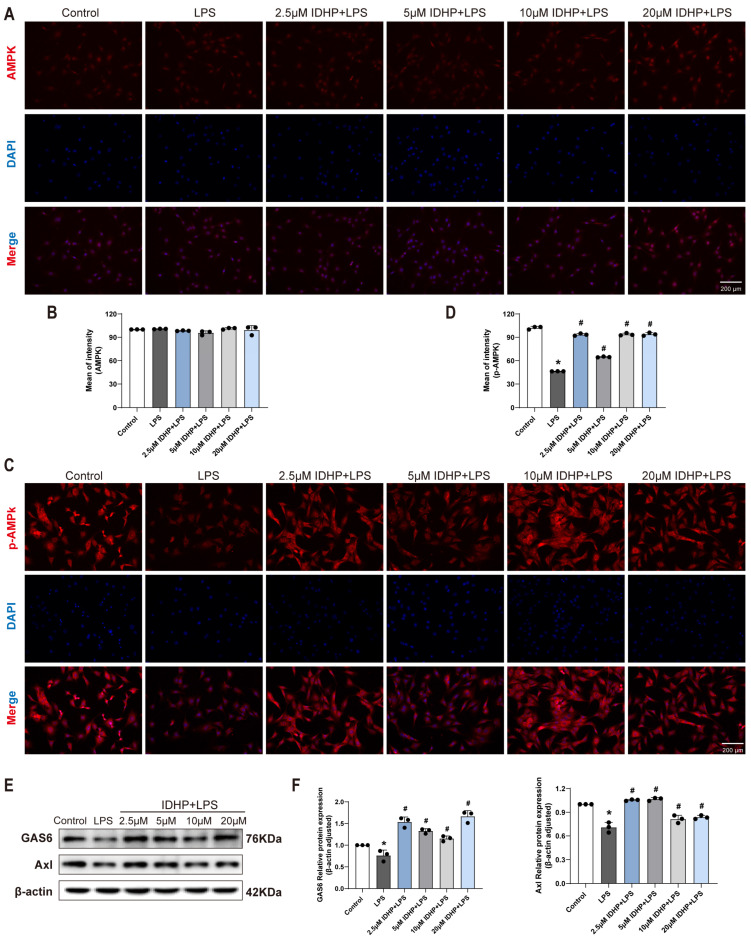
IDHP ameliorates LPS-induced cardiomyocyte injury by activating GAS6/Axl signaling axis and its downstream effector AMPK. (**A**,**B**) Immunofluorescence staining and corresponding statistical analysis of AMPK in HL-1 cells, n = 3. (**C**,**D**) Immunofluorescence staining and corresponding statistical analysis of p-AMPK in HL-1 cells, n = 3. (**E**) Representative images of GAS6 and Axl were detected by Western blot. (**F**) Quantitative analysis of Western blots was normalized to β-actin, n = 3. * *p* < 0.05, vs. control group; ^#^ *p* < 0.05, vs. LPS group.

To elucidate the regulatory mechanism of IDHP on the GAS6/Axl-AMPK pathway in LPS-induced cardiomyocyte injury, mouse cardiomyocytes were analyzed using immunofluorescence staining and Western blotting. The experimental results (Figure 4A–D and Figure 5A–D) showed that compared to the control group, LPS stimulation significantly decreased the fluorescence intensity of GAS6, Axl, and p-AMPK (*p* < 0.05). Notably, IDHP treatment (2.5–20 μM) significantly elevated the fluorescence signals of these proteins (*p* < 0.05). Western blot analysis (Figure 5E,F) further confirmed that IDHP significantly reversed the LPS-induced downregulation of GAS6 and Axl protein expression (*p* < 0.05). Collectively, these findings indicate that IDHP exerts its protective effect against LPS-induced cardiomyocyte injury by activating the GAS6/Axl signaling axis and promoting AMPK phosphorylation. This demonstrates that the GAS6/Axl-AMPK pathway is the key molecular mechanism underlying its cardioprotective efficacy.

### 2.5. GAS6 KO Reverses the Protective Effect of IDHP Against LPS-Induced Cardiomyocyte Injury

**Figure 6:**
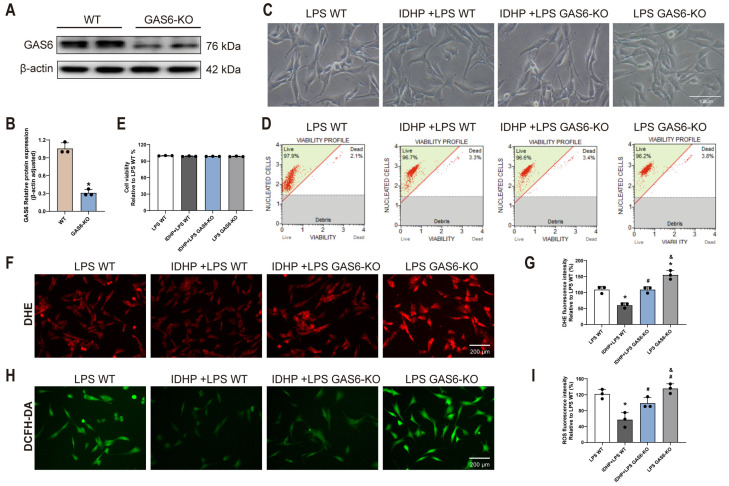
GAS6 kockout reverses the protective effect of IDHP against LPS-induced cardiomyocyte injury. (**A**) Representative images of GAS6 were detected by Western blot. (**B**) Quantitative analysis of Western blots was normalized to β-actin. (**C**) Typical morphological diagram of HL-1 cells in different groups. (**D**,**E**) Typical cell viability plots of HL-1 cells in different groups and corresponding statistical graph, n = 3. (**F**,**G**) DHE staining and statistical plots in HL-1 cells, n = 3. (**H**,**I**) DCFH-DA staining and statistical plots in HL-1 cells, n = 3. * *p* < 0.05, vs. LPS WT group; ^#^ *p* < 0.05, vs. IDHP + LPS WT group; ^&^ *p* < 0.05, vs. IDHP + LPS GAS6-KO group.

To elucidate the role of GAS6 in IDHP-mediated protection against LPS-induced cardiomyocyte injury, we generated a GAS6-knockout (GAS6-KO) cardiomyocyte cell line. Successful knockout was confirmed by Western blot analysis (*p* < 0.05, Figure 6A,B). Additionally, cell morphology and viability assays demonstrated that GAS6 knockout did not significantly alter the basal state of cardiomyocytes (Figure 6C–E). Subsequently, oxidative stress levels were assessed using DHE and DCFH-DA fluorescent probes (*p* < 0.05, Figure 6F,I). Results revealed that compared to the LPS-treated wild-type (WT) group and the LPS-treated GAS6-KO group, the IDHP-treated group exhibited significantly reduced DHE fluorescence intensity (*p* < 0.05), indicating effective suppression of ROS accumulation by IDHP. In contrast, GAS6-KO cardiomyocytes displayed markedly enhanced DHE fluorescence intensity relative to both the LPS WT group and the IDHP + LPS WT group (*p* < 0.05), demonstrating that GAS6-KO reversed IDHP’s protective effect and exacerbated ROS accumulation. Consistent results were further validated using DCFH-DA detection. These results provide clear evidence that GAS6 is an essential molecule for IDHP to counteract LPS-induced oxidative stress injury in cardiomyocytes. GAS6 deficiency not only aggravates intracellular redox imbalance but also reverses the antioxidative protective effects of IDHP.

## 3. Discussion

Sepsis refers to life-threatening organ dysfunction caused by a dysregulated host response to infection, with the heart being one of the most severely affected organs. The incidence of septic myocardial injury reaches 40–50% among sepsis patients [1,2]. Clinically, it often manifests as left ventricular dilation, normal or reduced filling pressure, decreased left ventricular ejection fraction, and ventricular dysfunction [28,29]. Despite diverse existing clinical therapeutic strategies, mortality of septic myocardial injury remains high. This underscores the urgency and importance of in-depth exploration into its pathogenesis, discovery of novel therapeutic targets, and development of safe and effective treatment options.

In recent years, our research team has focused on identifying specific drugs from traditional Chinese medicine to achieve effective treatment for septic myocardial injury. *Salvia miltiorrhiza* (Danshen) is commonly used clinically for cardiovascular diseases; for instance, Guanxin Danshen Dripping Pills treat coronary heart disease [30], and Yindan Xinnaotong Soft Capsules prevent atherosclerosis [31]. IDHP is an active ingredient screened among metabolites of various Danshen compound formulas by Professor Zheng Xiaohui. Previous studies revealed its pharmacological effects, including anti-inflammatory, anti-fibrotic, vasodilatory, anti-ischemia–reperfusion injury, and anti-aging properties [10,12,32]. Mechanistically, Wang et al. demonstrated in an isolated rat mesenteric artery experiment induced by norepinephrine that IDHP induces vasodilation by promoting K^+^ efflux to induce membrane hyperpolarization [33]. More importantly, in a rat myocardial ischemia–reperfusion injury (IRI) model, whether administered as a pretreatment or post-treatment strategy, IDHP consistently improved myocardial energy metabolism (increasing ATP production) and protected myocardial ultrastructure. Building on these findings, this study systematically elucidates that IDHP exerts significant protective effects against LPS-induced cardiomyocyte injury by activating the GAS6/Axl-AMPK signaling pathway.

Oxidative stress plays a critical role in the pathogenesis of sepsis [34]. Clinical studies reveal significant differences in antioxidant capacity between survivors and non-survivors of sepsis: although survivors exhibit reduced plasma antioxidant levels during the early disease phase, they demonstrate rapid recovery or even surpass normal levels during convalescence [35]. This phenomenon indicates that oxidative stress is closely associated with sepsis-induced organ dysfunction, making therapeutic strategies targeting antioxidant pathways a highly promising research direction. At the molecular level, the Nrf2 transcription factor serves as the central regulator of cellular responses to oxidative stress. By activating the expression of multiple antioxidant genes, Nrf2 effectively clears ROS and suppresses inflammatory cascades, thereby mitigating oxidative stress damage in septic cardiomyocytes [36]. Research demonstrates that dysregulation of the Nrf2 signaling pathway in septic myocardial injury exacerbates mitochondrial dysfunction and cellular pyroptosis, while specific activation of this pathway significantly ameliorates myocardial damage [37]. The key innovation of this study lies in confirming that IDHP potently activates the Nrf2 transcription factor and its downstream antioxidant defense system. This mechanism significantly reduces intracellular ROS levels in cardiomyocytes, establishing a novel therapeutic target for myocardial protection in sepsis.

GAS6 functions as a pleiotropic molecule involved in multiple disease processes, playing a crucial role in the onset and progression of cardiovascular diseases, cancer, and diabetes [38,39]. Notably, GAS6 expression in cardiac tissue exhibits significant developmentally specific patterns, with levels markedly elevated in the postnatal heart compared to the fetal period, suggesting its potential role in maintaining postnatal cardiac homeostasis [40]. Studies have found that plasma GAS6 concentrations are elevated in patients with sepsis and septic shock, positively correlating with disease severity [41]. This evidence indicates that GAS6 plays an important role in the pathological mechanisms of septic myocardial injury.

AMPK, a heterotrimeric protein ubiquitously present in eukaryotic cells, functions as the “central regulatory switch” for energy metabolism by sensing intracellular energy fluctuations to regulate glucose and lipid metabolism [42]. Recent studies have demonstrated that pharmacological or small-molecule compound-mediated activation of AMPK significantly suppresses experimental sepsis in mouse models, indicating its protective role in the pathogenesis of this disease [43,44]. Notably, GAS6 has been reported to attenuate vascular calcification and hyperlipidemia through modulation of the AMPK pathway [45].

Therefore, this study focuses on whether the GAS6/Axl and AMPK signaling pathways contribute to the cardioprotective effects of IDHP. This study innovatively discovered that IDHP activates the core metabolic regulator AMPK by restoring the suppressed GAS6/Axl signaling pathway, achieving multi-level cytoprotective effects: (1) significantly reducing intracellular ROS levels by activating the Nrf2 transcription factor and its regulated antioxidant defense system; (2) effectively inhibiting apoptosis by modulating the expression of Bax; (3) blocking the cytokine storm by suppressing pro-inflammatory factor secretion (IL-6, TNF-α) and regulating the CCL2/CCR2 chemokine axis. Crucially, in GAS6-knockout cell models, IDHP’s protective effects were significantly reversed. This key evidence not only confirms GAS6/Axl as the essential target for IDHP’s cardioprotection but also clarifies the molecular mechanism by which this signaling axis exerts protection via AMPK activation.

This study confirms IDHP’s protective effect against oxidative damage in cardiomyocytes through in vitro experiments but acknowledges certain limitations. Firstly, future research should validate its mechanism via post-conditioning experiments. Secondly, given that septic myocardial injury is often accompanied by endothelial cell dysfunction, subsequent studies could extend to endothelial cell models to explore IDHP’s multi-target protective effects. Additionally, systematic pharmacokinetic studies on IDHP—including tissue distribution characteristics and metabolite analysis—are recommended. This would provide crucial theoretical foundations for improving its pharmacodynamic and pharmacokinetic evaluation systems as well as toxicological safety assessments, thereby advancing the clinical translation of IDHP for treating septic myocardial injury.

## 4. Materials and Methods

### 4.1. Cell Culture and Treatment

HL-1 cells were cultured in Claycomb Medium supplemented with 10% fetal bovine serum (FBS) and 1% penicillin/streptomycin at 37 °C under 5% CO_2_. Passaging was performed using 0.25% trypsin-EDTA digestion when cells reached 80% confluence. Cells were cryopreserved in freezing medium (90% FBS + 10% DMSO) and stored in liquid nitrogen. An LPS injury model was established by treating cells with 10 μg/mL LPS for 6 h. IDHP (2.5–50 μM) was added 3 h before LPS treatment, while the control group received 0.1% DMSO.

### 4.2. Experimental Design

IDHP is an active compound identified by Professor Xiaohui Zheng’s research group (our research team) from the metabolites of Danshen compounds. IDHP was synthesized according to the method described by Bai et al. [12,46]. Its molecular formula is C_12_H_16_O_5_, with a molecular weight of 240.1. The IDHP sample used in this experiment had a purity of >99.9%.

IDHP Toxicity Assay: (1) Groups: Control group and IDHP groups (2.5, 5, 10, 20, and 50 µM IDHP). (2) Treatment: Control group received DMSO; IDHP groups were treated with IDHP for 24 h before analysis.

IDHP Protection Against LPS Injury: (1) Groups: Control, LPS, and IDHP pretreatment (2.5, 5, 10, 20 µM) + LPS groups. (2) Treatment: Control and LPS groups received DMSO; IDHP pretreatment groups were incubated with IDHP for 3 h. All groups (except control) were then exposed to 10 μg/mL LPS for 6 h.

GAS6-KO Mouse Cardiomyocyte Assay: (1) Groups: LPS WT; IDHP (10 μM) + LPS WT; IDHP (10 μM) + LPS GAS6-KO; LPS GAS6-KO. (2) Treatment: LPS WT and LPS GAS6-KO groups received DMSO; IDHP + LPS groups were pretreated with 10 μM IDHP for 3 h before LPS injury (6 h).

### 4.3. The GAS6-KO Mouse Cardiomyocyte Line

The GAS6-KO recombinant vector was purchased from Xi’an GeneBio Biotechnology Co., Ltd. (Xi’an, China). A dual-gRNA/Cas9 expression vector was constructed using the PX459 backbone via dual-gRNA fragment cloning technology. Mouse cardiomyocytes at 60% confluence were transfected with 0.8 μg/mL GAS6-KO plasmid and 1 μL liposome (100 μL transfection mixture, incubated for 20 min at room temperature). After 48 h, puromycin was added to the medium and refreshed every 2 days to establish the GAS6-KO cell line.

### 4.4. Cell Viability and ROS Detection

Cell viability was determined using the Muse™ Count & Viability Kit (Merck, Darmstadt, Germany), where cells were stained and incubated in the dark for 5 min prior to analysis on the Muse Cell Analyzer (Merck, Darmstadt, Germany). For ROS detection, cells were incubated with either 5 μM dihydroethidium (DHE) or 5 μM 2′,7′-dichlorodihydrofluorescein diacetate (DCFH-DA) in the dark for 30 min at 37 °C. Fluorescence images were captured using an Invitrogen EVOS M5000 microscope (Thermo Fisher Scientific, Waltham, MA, USA), and fluorescence intensity was quantified with ImageJ software 1.53e (National Institutes of Health, Bethesda, MD, USA).

### 4.5. Immunofluorescence Staining

Cells were fixed with 4% paraformaldehyde (15 min, room temperature), permeabilized with 0.3% Triton X-100 (10 min), and blocked with goat serum. Primary antibodies (anti-GAS6, anti-Axl, anti-Bax, anti-Nrf2, anti-AMPK, anti-p-AMPK; Table 1) were applied overnight at 4 °C. After PBS washing, fluorophore-conjugated secondary antibodies and DAPI were added (1 h, dark). Images were acquired using a Nikon Eclipse C1 microscope (Nikon, Tokyo, Japan) and analyzed with ImageJ.

### 4.6. Western Blot

Cells were lysed in RIPA buffer (30 min, ice) and centrifuged (12,000 rpm, 5 min, 4 °C). Protein concentration was determined via BCA kit (Beyotime, Shanghai, China), mixed with 5 × loading buffer (4:1 ratio), and denatured (100 °C, 5 min). Proteins (10–15 μg) were separated by 8%/10% SDS-PAGE, transferred to PVDF membranes (Millipore, Darmstadt, Germany), blocked with 5% skim milk, and incubated with primary antibodies (4 °C, overnight). HRP-conjugated secondary antibodies (biosharp, Hefei, China) were applied (1 h, room temperature), and signals were detected using a MiNiChemi610 system (SinSage Technology Co., Ltd., Beijing, China). Band intensity was quantified by ImageJ (β-actin as loading control).

### 4.7. RNA Extraction, RT, and qPCR

Total RNA was extracted using TRIzol (Accura, Taicang, China) and reverse-transcribed with HiFiScript gDNA Removal RT MasterMix (CWBIO, Taizhou, China). qPCR was performed using SuperStar Universal SYBR MasterMix (CWBIO). Primer sequences are listed in Table 2. Cycling conditions: 95 °C for 10 min; 40 cycles of 95 °C for 5 s and 60 °C for 30 s; then 94 °C for 30 s, 60 °C for 90 s, and 94 °C for 10 s. mRNA levels were normalized to ACTB.

### 4.8. Statistical Analysis

Data are expressed as the mean ± SD. Student’s *t*-test or one-way ANOVA with Tukey’s HSD post hoc test was performed using GraphPad Prism 9 (USA). *p* < 0.05 was considered statistically significant.

The catalog numbers of the reagents are provided in Supplementary Table S1.

## 5. Conclusions

**Figure 7:**
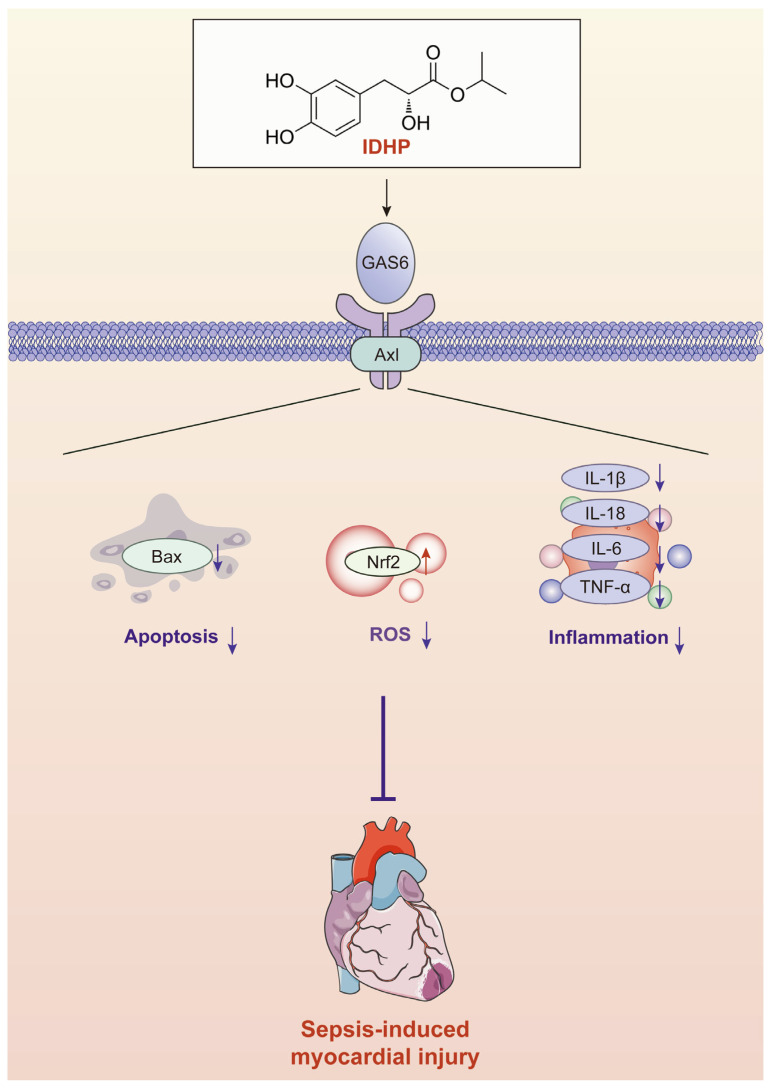
Schematic diagram depicting the molecular mechanism of IDHP in alleviating myocardial cell injury through regulating the GAS6/Axl-AMPK signaling pathway.

In summary, this study demonstrates that IDHP exerts definite cardioprotective effects and elucidates the positive role of the GAS6/Axl-AMPK signaling pathway (Figure 7). Furthermore, IDHP inhibits apoptosis, mitochondrial dysfunction, inflammatory responses, and oxidative stress induced by cardiomyocyte injury. These findings highlight the significant therapeutic potential of targeting the GAS6/Axl-AMPK signaling pathway and provide a theoretical basis for investigating the mechanisms of active ingredients in TCM compound metabolites and developing IDHP as a clinical treatment for septic myocardial injury.

## Figures and Tables

**Table 1:** The manufacturer and product number of experimental antibodies.

Experimental Antibodies	Manufacturers	Product Numbers
GAS6	Servicebio, Wuhan, China	GB11636
Axl	Bioss, Woburn, MA, USA	bs-5180R
Bax	Bioss, Woburn, MA, USA	bs-0127R
Nrf2	Boster Biological Technology, Pleasanton, CA, USA	A00078-1
AMPK	Abcam, Cambridge, UK	ab110036
p-AMPK	Bimake, Houston, TX, USA	A5740

**Table 2:** PCR primer sequences.

Primer Names	Primer Sequence
mouse-TNF-α	F: 5′-GGTGCCTATGTCTCAGCCTCTT-3′ R: 5′-GCCATAGAACTGATGAGAGGGAG-3′
mouse-IL-6	F: 5′-TACCACTTCACAAGTCGGAGGC-3′ R: 5′-CTGCAAGTGCATCATCGTTGTT-3′
mouse-IL-1β	F: 5′-TGGACCTTCCAGGATGAGGACA-3′ R: 5′-GTTCATCTCGGAGCCTGTAGTG-3′
mouse-IL-18	F: 5′-TCAAAGTGCCAGTGAACCC-3′ R: 5′-TGTCTGATTCCAGGTCTCCA-3′
mouse-CCL25	F: 5′-TGGAGGATGGGAGGAGTC-3′ R: 5′-TGGTGGGTCTGGTCTTGT-3′
mouse-CCR9	F: 5′-TCTGCATTACCATCTGGGTGA-3′ R: 5′-ATTCCCCACTGACTTGACTGT-3′
mouse-CCL2	F: 5′-CCTGCTGCTACTCATTCACCA-3′ R: 5′-ATTCCTTCTTGGGGTCAGCA-3′
mouse-CCR2	F: 5′-AAACGTCTCTGCAAACAGTGC-3′ R: 5′-CAACCGAGACCTCTTGCTCC-3′
mouse-β-actin	F: 5′-GGCTGTATTCCCCTCCAATCG-3′ R: 5′-CCAGTTGGTAACAATGCCATGT-3′

## Data Availability

Data presented in this study is contained within the article and Supplementary Material. Further inquiries can be directed to the corresponding author.

## Supplementary Materials

The following supporting information can be downloaded at: https://www.mdpi.com/article/10.3390/ph18081188/s1, Table S1: Experimental reagent information table.
