# Supplementary material for: IDHP Mitigates LPS-Induced Cardiomyocyte Injury via the GAS6/Axl-AMPK Axis: A Multi-Target Strategy Counteracting Inflammation, Oxidative Stress, and Apoptosis
Source: Pharmaceuticals (Basel). 2025 Aug 12;18(8):1188. doi: 10.3390/ph18081188 (PMC12388950; doi:10.3390/ph18081188)
Supplement: Supplementary file 1 [file pharmaceuticals-18-01188-s001.zip › pharmaceuticals-3572681-supplementary.pdf]

Supplementary Table S1. Experimental Reagent Information Table.

**Experimental Reagent**	**Manufacturers**	**Product Numbers**
FBS	Cell-Box	AUS-01S-02
EDTA	Xi'an Guoan Biotechnology Co., Ltd	C10H16N208
LPS	SIGMA	L2880-100MG
DMSO	Sangon Biotech	A610163
liposome	invitrogen	11668019
DHE	Beyotime	S0063
DCFH-DA	Beyotime	S0033S-1
4% paraformaldehyde	Sangon Biotech	E672002-0500
Triton	Sangon Biotech	A600198-0500
DAPI	biosharp	BL739B
TRIzol	Accurate Biotechnology	AG21102
HiFiScript gDNA Removal RT MasterMix	CWBIO	CW2020M
SuperStar Universal SYBR Master Mix	CWBIO	CW3360M
penicillin/streptomycin	Proteintech	PR40022
